# Multi-component radiological model based on intratumoral CT threshold segmentation for predicting visceral pleural invasion in lung adenocarcinoma ≤ 30 mm

**DOI:** 10.1186/s13244-026-02351-z

**Published:** 2026-07-24

**Authors:** Yuanxin Sun, Jing Chen, Tingting Wang, Lu Zhang, Tingjia Xue, Weiqiu Jin, Hong Yu, Xiaodan Ye

**Affiliations:** 1 Department of Radiology, Shanghai Chest Hospital, Shanghai Jiao Tong University School of Medicine, Shanghai, China; 2 Shanghai Institute of Medical Imaging, Shanghai, China; 3 Department of Radiology, Zhongshan Hospital, Fudan University, Shanghai, China; 4 Department of MRI, Jiaozuo People’s Hospital, Jiaozuo, China

**Keywords:** Adenocarcinoma of lung, Deep learning, Radiomics, Tomography (X-ray computed), Visceral pleural invasion

## Abstract

**Objectives:**

This retrospective study aims to investigate the value of intratumoral computed tomography (CT) threshold segmentation in radiomics, deep learning (DL), and radiomics-DL combined models for predicting visceral pleural invasion (VPI) in lung adenocarcinoma (LUAD) ≤ 30 mm.

**Materials and methods:**

Patients with invasive LUAD who underwent surgery and had preoperative thin-slice CT scans within four weeks were enrolled from two centers (*n* = 816). Patients from center 1 were divided into a training set (TS, *n* = 591) and an internal test set (ITS, *n* = 98) based on surgical time. Patients from center 2 constituted the external test set (ETS, *n* = 127). Solid, ground-glass, and peritumoral components were extracted using intratumoral CT threshold segmentation and peritumoral expansion methods. The radiomics model was a Random Forest Classifier; the DL model was a pre-trained Vision Transformer (ViT) fine-tuned on three components; the combined model integrated features from two pipelines. Model performance was evaluated by the area under the receiver operating characteristic curve (AUC), sensitivity, and specificity. Clinical utility and model interpretability were evaluated using decision curve analysis and SHapley Additive exPlanations (SHAP), respectively.

**Results:**

Radiomics, ViT, and radiomics-ViT models achieved AUCs of 0.865, 0.858, and 0.895 in TS; 0.865, 0.844, and 0.852 in ITS; and 0.844, 0.816, and 0.823 in ETS, respectively. Radiomics-ViT model achieved the highest sensitivity, with ViT features contributing the most.

**Conclusion:**

A hybrid multi-component feature pipeline could serve as a reliable and highly sensitive tool for VPI prediction in LUAD ≤ 30 mm.

**Critical relevance statement:**

The multi-component radiomics-ViT model achieved high sensitivity for VPI prediction in LUAD ≤ 30 mm, which is a promising tool for preoperative treatment design and prognostic assessment.

**Key Points:**

- CT attenuation-defined components remain underexplored in artificial intelligence (AI) models for VPI prediction.
- Solid, ground-glass, and peritumoral components enable AI models for VPI prediction.
- Multi-component inputs effectively capture tumor heterogeneity for VPI prediction in LUAD ≤ 30 mm.

**Graphical Abstract:**

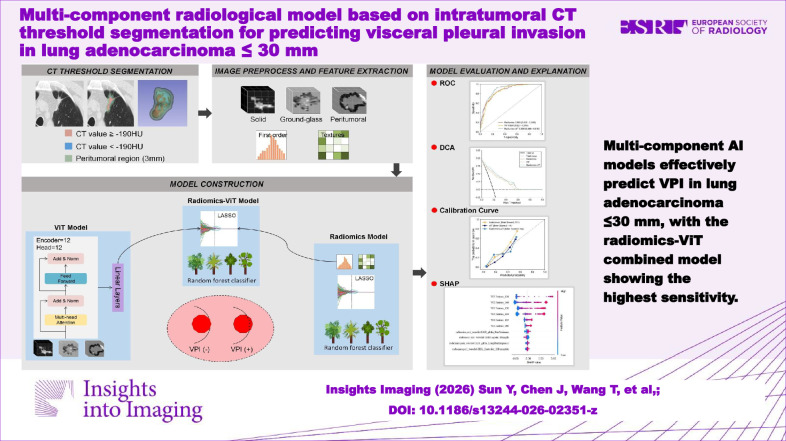

## Introduction

Lung cancer is the most common and lethal malignancy worldwide [1], with lung adenocarcinoma (LUAD) being the most prevalent histological type. Visceral pleural invasion (VPI), defined as tumor cell invasion beyond the pleural elastic layer, is an important pathological and prognostic factor in LUAD. VPI has been incorporated into the T category [2], thereby upstaging tumors ≤ 30 mm from stage IA to IB. It is associated with significantly worse overall survival and disease-free survival, even in early-stage tumors [3, 4]. For tumors ≤ 30 mm with VPI, systematic lymphadenectomy and lobectomy are recommended surgical approaches [5, 6]. For stage IB patients following radical surgery, adjuvant chemotherapy is considered for those with VPI after multidisciplinary team evaluation [7]. Therefore, timely preoperative detection of VPI is essential for optimizing surgical planning and the initiation of adjuvant therapy.

The gold standard for diagnosing VPI is pathological confirmation of tumor invasion beyond the elastic fiber layer; however, it cannot be performed intraoperatively due to the time-consuming nature of elastic fiber staining. Alternative techniques such as autofluorescence and confocal laser endomicroscopy also remain under investigation [8, 9]. Consequently, CT imaging serves as a crucial noninvasive tool for preoperative VPI assessment. Certain semantic CT features, such as the jellyfish sign [10], have been identified as reliable indicators of VPI but are subject to observer variability. Advances in artificial intelligence, including radiomics and deep learning (DL) [11, 12] enable quantitative and objective analysis of the entire tumor. These approaches demonstrate strong reproducibility and diagnostic efficiency. Furthermore, the popularity of Natural Language Processing and its Transformer architectures has introduced Vision Transformer (ViT) to image classification, which captures global structural context and spatial dependencies [13].

However, previous studies constructed models based on segmentation of the entire tumor, thereby treating it as a single region without explicitly accounting for intratumoral heterogeneity [14–17]. Pure ground-glass, subsolid, and solid components demonstrate distinct biological behaviors [18]. Compared to subsolid nodules, solid tumors are more strongly associated with lymphatic, vascular, and pleural invasion [19]. In addition, peritumoral features play a crucial role in VPI prediction [20].

Thus, the primary aim of this study is to develop and evaluate multi-component radiomics, ViT, and radiomics-ViT combined models for VPI prediction, supplemented by intratumoral threshold segmentation and peritumoral expansion.

## Materials and methods

### Patient selection

This retrospective study was approved by the institutional review board of Zhongshan Hospital, Fudan University (B2021-128) and Shanghai Chest Hospital, Shanghai Jiao Tong University School of Medicine (IS23088). The requirement for obtaining informed consent has been waived.

**Fig. 1:**
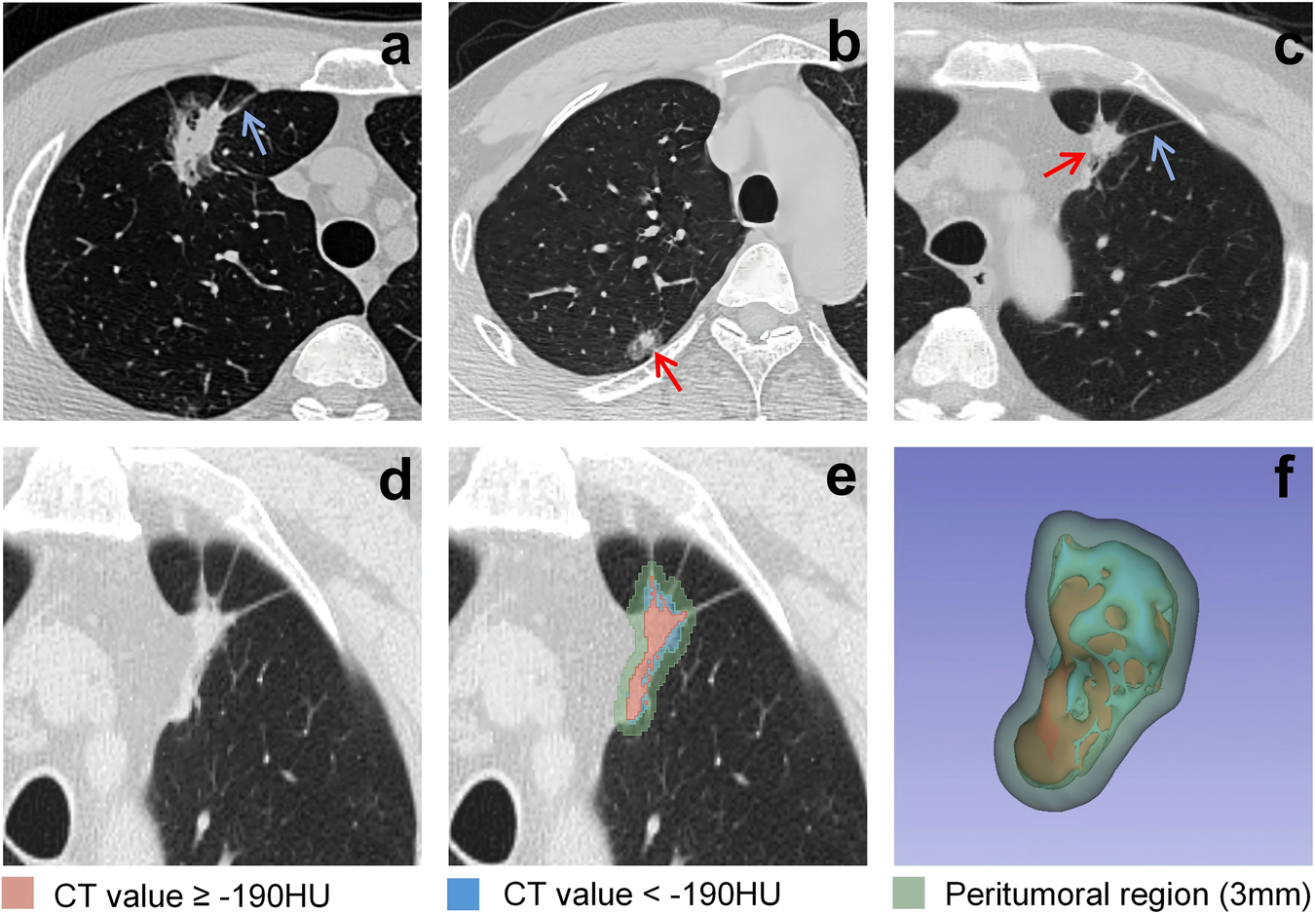
Illustration of pleural invasion and CT threshold segmentation. **a** Pleural tag (blue arrow). **b** Pleural attachment (red arrow). **c** Coexistence of pleural tag and pleural attachment (blue and red arrows). **d** Axial CT image. **e** Threshold segmentation demonstration. **f** 3D visualization of the tumor

**Fig. 2:**
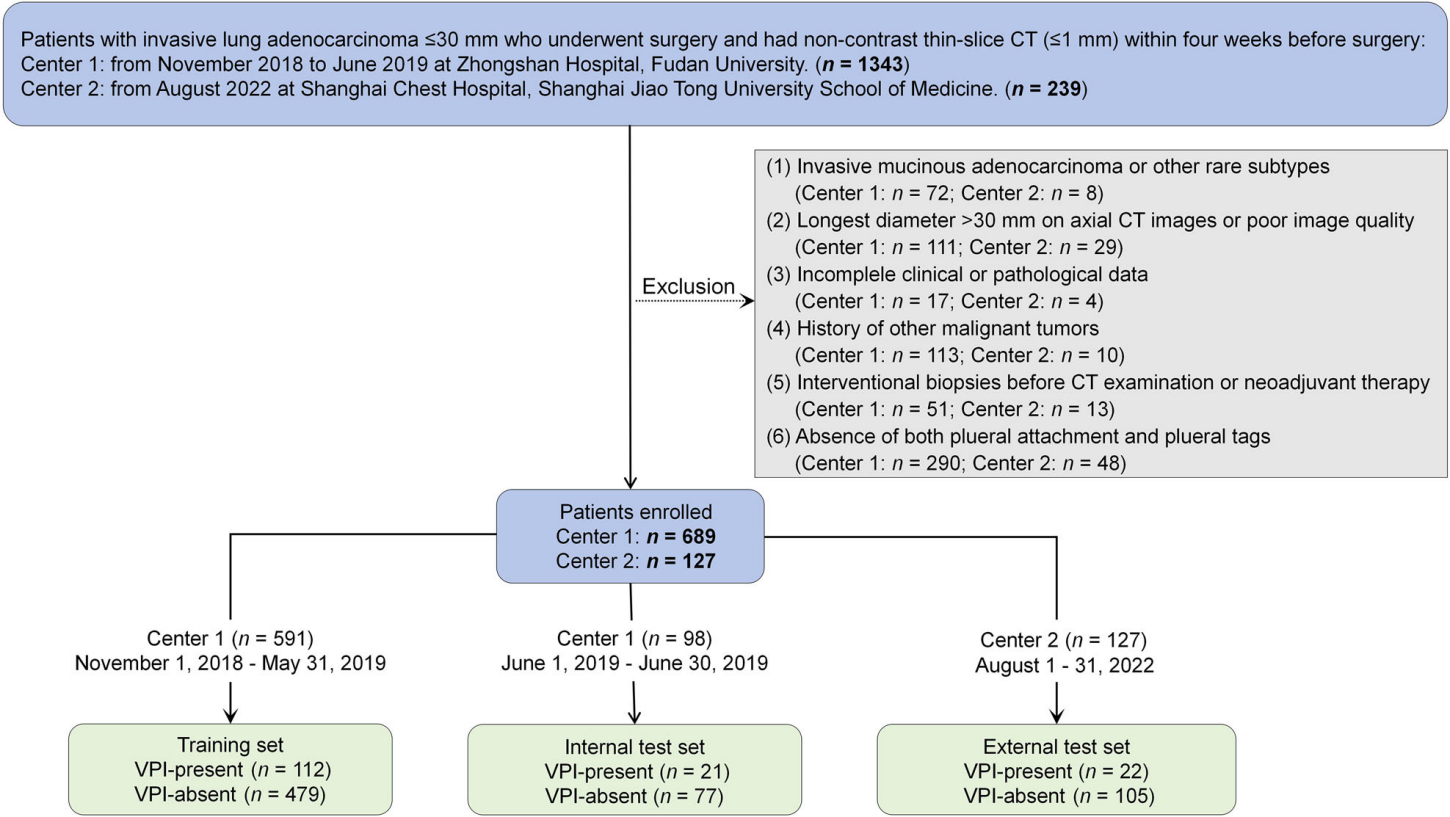
Flowchart of patient inclusion and exclusion

This dual-center study initially included 1582 patients with LUAD who underwent surgical treatment: 1343 patients from Zhongshan Hospital, Fudan University (November 1, 2018–June 30, 2019) and 239 patients from Shanghai Chest Hospital, Shanghai Jiao Tong University School of Medicine (August 1–31, 2022). All participants underwent preoperative non-contrast thin-slice (≤ 1 mm) CT scans within four weeks before surgery. After reviewing all cases, patients were excluded if they met any of the following criteria: (1) mucinous adenocarcinoma or other rare subtypes; (2) longest diameter > 30 mm on axial CT images or poor image quality; (3) incomplete clinical or pathological data; (4) history of other malignant tumors; (5) interventional biopsies before CT examination or neoadjuvant therapy; (6) absence of both pleural attachment and pleural tags (Fig. 1) [21]. Pleural attachment and tags were independently assessed on axial, coronal, and sagittal CT images on the lung window (window width: 1500 HU; window level: −400 HU) by two radiologists with 5 and 10 years of experience, respectively. In cases of disagreement, a senior radiologist with 20 years of experience made the final decision. Finally, a total of 816 patients were enrolled, with the detailed inclusion and exclusion process illustrated in Fig. 2. Patients from center 1 were divided into a training set (591) and an internal test set (98) according to surgical time. Patients from center 2 constituted the external test set (127).

### CT imaging acquisition and pathological evaluation

All patients underwent non-contrast thin-slice (≤ 1 mm) chest CT scans within four weeks before surgery. Scans were performed in the supine position during breath-holding. Reconstruction was performed using a bone or a standard imaging algorithm, with an axial slice thickness of 0.625 or 1 mm, according to the scanning protocols of the respective CT scanners. A variety of multi-detector CT scanners were utilized in two centers, including 16- to 512-slice CT scanners from Siemens Medical, GE Healthcare, United Imaging Healthcare, Canon Medical Systems, and Philips. Detailed scanning parameters are provided in Supplementary Tables S1 and S2. Pathological evaluation was performed by two pathologists using elastic fiber staining. Patients with disrupted elastic fiber layers were classified as VPI-present, whereas those with intact layers were classified as VPI-absent.

### Nodule delineation and threshold segmentation

All CT images were imported into 3D Slicer (version 5.6.2; https://www.slicer.org) for manual delineation, an open-source medical image processing software. Segmentation was performed primarily on axial images using the lung window (window width: 1500 HU; window level: -400 HU), with the mediastinal window (window width: 350 HU; window level: 40 HU) and multiplanar reformations used when necessary. A primary radiologist with 5 years of experience delineated regions of interest (ROIs) layer by layer, avoiding large blood vessels and bronchi adjacent to the tumor. Subsequently, a senior radiologist with 10 years of experience reviewed all ROIs and randomly re-delineated 30 lesions. Inter-observer reproducibility of the radiomics features was assessed by calculating intraclass correlation coefficients (ICCs) from lesions independently delineated by two observers. After ROI delineation, a threshold of -190 HU was applied to segment the original tumor ROI into two subregions: the solid component (≥ −190 HU) and the ground-glass component (< −190 HU). Subsequently, a peritumoral component was created by expanding the tumor boundary outward by 3 mm in all directions. The threshold segmentation process is illustrated in Fig. 1. All original images and segmentation masks were resampled to an isotropic voxel size of 0.8 × 0.8 × 0.8 mm³.

### Radiomics model construction

Radiomics features were respectively extracted from solid, ground-glass, and peritumoral ROIs using SlicerRadiomics (Version:8426cdf), an extension of 3D Slicer that provides an interface to the PyRadiomics library and is developed and maintained by the Artificial Intelligence in Medicine Program, Harvard Medical School. Data were discretized using a bin width of 25 HU and normalized by *Z*-score. In total, 107 original features were extracted for each ROI, including 14 shape features, 18 first-order statistical features, 24 gray level co-occurrence matrix (GLCM) features, 14 gray level dependence matrix (GLDM) features, 16 gray level run length matrix (GLRLM) features, 16 gray level size zone matrix (GLSZM) features, and 5 neighboring gray tone difference matrix (NGTDM) features. Additionally, wavelet transforms generated 744 features using eight filter combinations (LLH, LLL, HHH, HHL, HLL, HLH, LHL, LHH).

Feature selection was performed on the training set. First, irrelevant features were removed using variance analysis and mutual information. Then, the Least Absolute Shrinkage and Selection Operator (LASSO) was applied to identify the most informative features. Based on the selected features, a random forest classifier was constructed using Scikit-learn (version 1.2.2). Five-fold cross-validation was employed with area under the curve (AUC) as the optimization target. Bayesian optimization was performed using Optuna (version 3.1.0) for selecting the best parameters.

### ViT model construction

A pre-trained three-dimensional (3D) ViT backbone was employed (https://github.com/AliAfs/SiT_3D/tree/main), with additional input branches and fusion layers tailored for this task. The pretrained model consists of twelve sequential encoder layers with twelve attention heads. Volumes from solid, ground-glass, and peritumoral ROIs were extracted and served as three separate inputs. During transfer learning, the first eleven encoder layers were frozen to preserve pretrained parameters for general feature extraction, while the final layer remained trainable for adaptation to VPI prediction. Training was conducted on an NVIDIA A100-80GB GPU using the Adam optimizer, with AUC as the objective metric. The model architecture is illustrated in Supplementary Fig. S1.

### Radiomics-ViT model construction

**Fig. 3:**
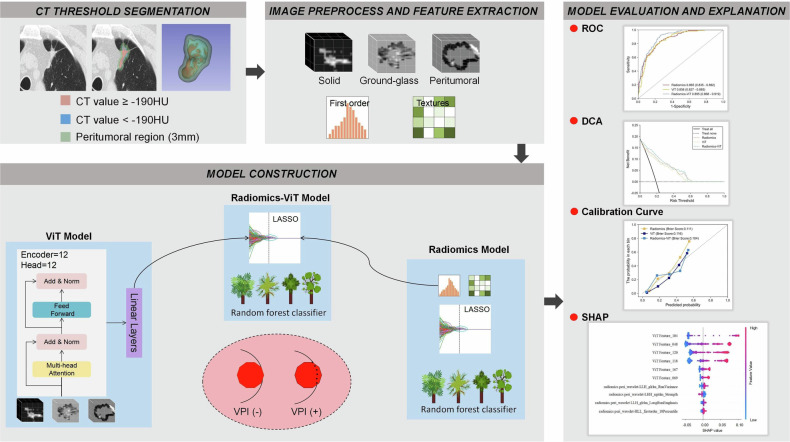
Data processing and modeling workflow

Finally, 256 ViT features were extracted and combined with radiomics features for feature selection and model construction, following the same procedures used for the radiomics model. Further interpretation of the combined model was performed using Shapley additive explanations (SHAP), a method based on Shapley values from cooperative game theory that quantifies each feature’s contribution to the prediction. The whole workflow is shown in Fig. 3.

### Statistical data analysis

Clinical and pathological data were analyzed using SPSS 27.0 software. Quantitative variables were first tested for normal distribution. Variables following a normal distribution were expressed as mean ± standard deviation ($\bar{x}\pm s$), while those not normally distributed were presented as median and interquartile range (IQR). Categorical variables were described using frequencies and proportions. Quantitative variables were compared using the two independent samples *t*-test, while categorical variables were compared using Pearson’s chi-square test or Fisher’s exact test. The DeLong test in MedCalc 23.0.9 software was employed to compare differences in AUC between models. Sensitivity, specificity, and decision curve analysis (DCA) were also performed to further evaluate the models. A two-sided *p* < 0.05 was considered statistically significant.

## Results

### Clinical and final pathological features

In all datasets, significant differences were observed between VPI-present and VPI-absent groups concerning the longest diameter, consolidation tumor ratio (CTR), Ki67, and high-grade histological patterns (*p* < 0.05). No significant statistical differences were found in age, gender, and smoking status within all datasets (*p* > 0.05). Clinical statistics are shown in Table 1.

**Table 1:** Clinical and pathological features of VPI-present and VPI-absent patients

Characteristic	Training set		*p* value	Internal test set		*p* value	External test set		*p* value
	VPI-present	VPI-absent		VPI-present	VPI-absent		VPI-present	VPI-absent	
*n*	112	479		21	77		22	105	
Clinical characteristics									
Age (y)^a^	62.0 (55.0, 67.0) [31.0–80.5]	61.0 (53.0, 67.0) [25.0–81.0]	0.071	61.0 (55.0, 64.0) [41.0–75.0]	61.0 (53.0, 66.0) [27.0–78.0]	0.700	66.0 (62.8, 70.0) [33.0–78.0]	61.0 (51.5, 70.0) [33.0–81.0]	0.127
Sex			0.077			0.400			0.057
Male	52 (46.4)	179 (37.4)		10 (47.6)	29 (37.7)		13 (59.1)	38 (36.2)	
Female	60 (53.6)	300 (62.6)		11 (52.4)	48 (62.3)		9 (40.9)	67 (63.8)	
Smoking			0.145			0.556			0.188
Formerly smoked or currently smokes	25 (22.3)	79 (16.5)		6 (28.6)	16 (20.8)		3 (13.6)	6 (5.7)	
Never smoked	87 (77.7)	400 (83.5)		15 (71.4)	61 (79.2)		19 (86.4)	99 (94.3)	
CT characteristics									
Longest diameter (mm)^a^	21.0 (15.0, 27.0) [4.0–30.0]	16.0 (12.0, 22.0) [5.0–30.0]	< 0.001	24.0 (20.0, 30.0) [15.0–30.0]	17.0 (12.0, 23.5) [7.0–30.0]	< 0.001	24.0 (19.0, 28.5) [12.0–30.0]	18.0 (14.0, 24.0) [8.0–30.0]	< 0.001
CTR^a^	0.96 (0.89, 0.98) [0.47–1.0]	0.88 (0.75, 0.95) [0.0–1.0]	< 0.001	0.98 (0.95, 0.99) [0.66–1.0]	0.89 (0.75, 0.94) [0–1.0]	< 0.001	0.97 (0.88, 1.0) [0.77–1.0]	0.77 (0.55, 0.91) [0–1.0]	< 0.001
Pathologically confirmed features									
Ki-67			< 0.001			< 0.001			0.030
< 30%	64 (57.1)	416 (86.8)		9 (42.9)	68 (88.3)		18 (81.8)	101 (96.2)	
≥ 30%	48 (42.9)	63 (13.2)		12 (57.1)	9 (11.7)		4 (18.2)	4 (3.8)	
HGPs			< 0.001			< 0.001			< 0.001
Present	82 (73.2)	122 (25.5)		16 (76.2)	24 (31.2)		17 (77.3)	34 (32.4)	
Absent	30 (26.8)	357 (74.5)		5 (23.8)	53 (68.8)		5 (22.7)	71 (67.6)	
LNM			< 0.001			0.173			0.003
N0	84 (75.0)	433 (90.4)		16 (76.2)	69 (89.6)		17 (77.3)	94 (89.5)	
Systematic lymph node dissection or sampling^c^	63 (75.0)^b^	331 (76.4)		15 (93.7)	54 (78.2)		11 (64.7)	50 (53.1)	
Selective lymph node sampling	21 (25.0)	102 (23.6)		1 (6.2)	15 (21.7)		6 (35.2)	44 (46.8)	
N1	10 (8.9)	18 (3.8)		1 (4.8)	1 (1.3)		0 (0.0)	2 (1.9)	
Systematic lymph node dissection or sampling	9 (90.0)	16 (88.9)		0 (0.0)	1 (100.0)		0 (0.0)	2 (100.0)	
Selective lymph node sampling	1 (10.0)	2 (11.1)		1 (100.0)	0 (0.0)		0 (0.0)	0 (0.0)	
N2	14 (12.5)	13 (2.7)		3 (14.3)	4 (5.2)		5 (22.7)	2 (1.9)	
Systematic lymph node dissection or sampling	11 (78.6)	12 (92.3)		3 (100.0)	4 (100.0)		4 (80.0)	1 (50.0)	
Selective lymph node sampling	3 (21.4)	1 (7.7)		0 (0.0)	0 (0.0)		1 (20.0)	1 (50.0)	
Nx (No lymph node sampling or dissection)	4 (3.6)	15 (3.1)		1 (4.8)	3 (3.9)		0 (0.0)	7 (6.7)	
STAS			0.049			0.054			0.173
Present	20 (17.9)	53 (12.0)		5 (23.8)	6 (7.8)		5 (22.7)	12 (11.4)	
Absent	92 (82.1)	426 (88.0)		16 (76.2)	71 (92.2)		17 (77.3)	93 (88.6)	
LVI			< 0.001			0.166			< 0.001
Present	16 (14.3)	17 (3.5)		3 (14.3)	4 (5.2)		7 (31.8)	6 (5.7)	
Absent	96 (85.7)	462 (96.5)		18 (85.7)	73 (94.8)		15 (68.2)	99 (94.3)	

*VPI* visceral pleural invasion, *CTR* consolidation tumor ratio, *LNM* lymph node metastasis, *STAS* spread through air spaces, *LVI* lymphovascular invasion, *HGPs* high-grade histological patterns including solid, micropapillary, and complex glandular patterns

^a^ Data are presented as the median, with the interquartile range (IQR) in parentheses and the range in square brackets

^b^ Percentages for each lymphadenectomy approach (systematic or selective) represent the proportion of patients within each nodal status (N0/N1/N2/Nx), rather than the overall group

^c^ Systematic lymph node dissection or sampling was defined as the sampling or removal of at least one N1 and three N2 stations, including the Subcarinal (station 7), based on the guidelines of the National Comprehensive Cancer Network (NCCN) and the International Association for the Study of Lung Cancer (IASLC)

### Model performance

The radiomics model, constructed with 15 selected features (Supplementary Table S3), was based on a random forest classifier and achieved AUCs of 0.865, 0.865, and 0.844 in the training, internal test, and external test sets, respectively. The best ViT model achieved AUCs of 0.858, 0.844, and 0.816 across the three datasets. The radiomics-ViT model utilized 19 selected features (Supplementary Table S4) and achieved AUCs of 0.895, 0.852, and 0.823 in the training, internal test, and external test sets, respectively. Table 2 summarizes the performance of all models, including sensitivity and specificity. Inter-observer consistency analysis indicated that the average ICC value for radiomics features was 0.930.

**Table 2:** Predictive performance of the radiomics, ViT, and radiomics–ViT models

Model	AUC	Sensitivity (%)	Specificity (%)
Training set			
Radiomics	0.865 (0.835–0.892)	83.0 (73.8–88.7)	74.7 (70.6–78.6)
ViT	0.858 (0.827–0.885)	78.6 (68.8–85.0)	79.1 (75.2–82.7)
Radiomics-ViT	0.895 (0.868–0.919)	92.8 (86.4–96.9)	73.3 (69.1–77.2)
Internal test set			
Radiomics	0.865 (0.781–0.925)	85.7 (63.7–97.0)	75.3 (64.2–84.4)
ViT	0.844 (0.757–0.910)	76.2 (52.8–91.8)	77.9 (67.0–86.6)
Radiomics-ViT	0.852 (0.766–0.916)	90.5 (69.6–98.8)	71.4 (60.0–81.2)
External test set			
Radiomics	0.844 (0.769–0.902)	90.9 (70.8–98.9)	60.9 (50.9–70.3)
ViT	0.816 (0.738–0.879)	72.7 (49.8–89.3)	70.5 (60.8–79.0)
Radiomics-ViT	0.823 (0.745–0.885)	95.4 (77.2–99.9)	60.0 (50.0–69.4)

Values in parentheses represent 95% confidence intervals (CIs)

*AUC* area under the curve

Subgroup analyses were performed based on tumor size, using a 20 mm cutoff commonly used in TNM staging and surgical decision-making. Tumors measuring 20–30 mm are associated with a higher risk of VPI than tumors ≤ 20 mm [22–24]. Therefore, model performance was separately evaluated in the ≤ 20 mm and 20–30 mm subgroups (Supplementary Fig. S2).

### Comparison of model performance

**Fig. 4:**
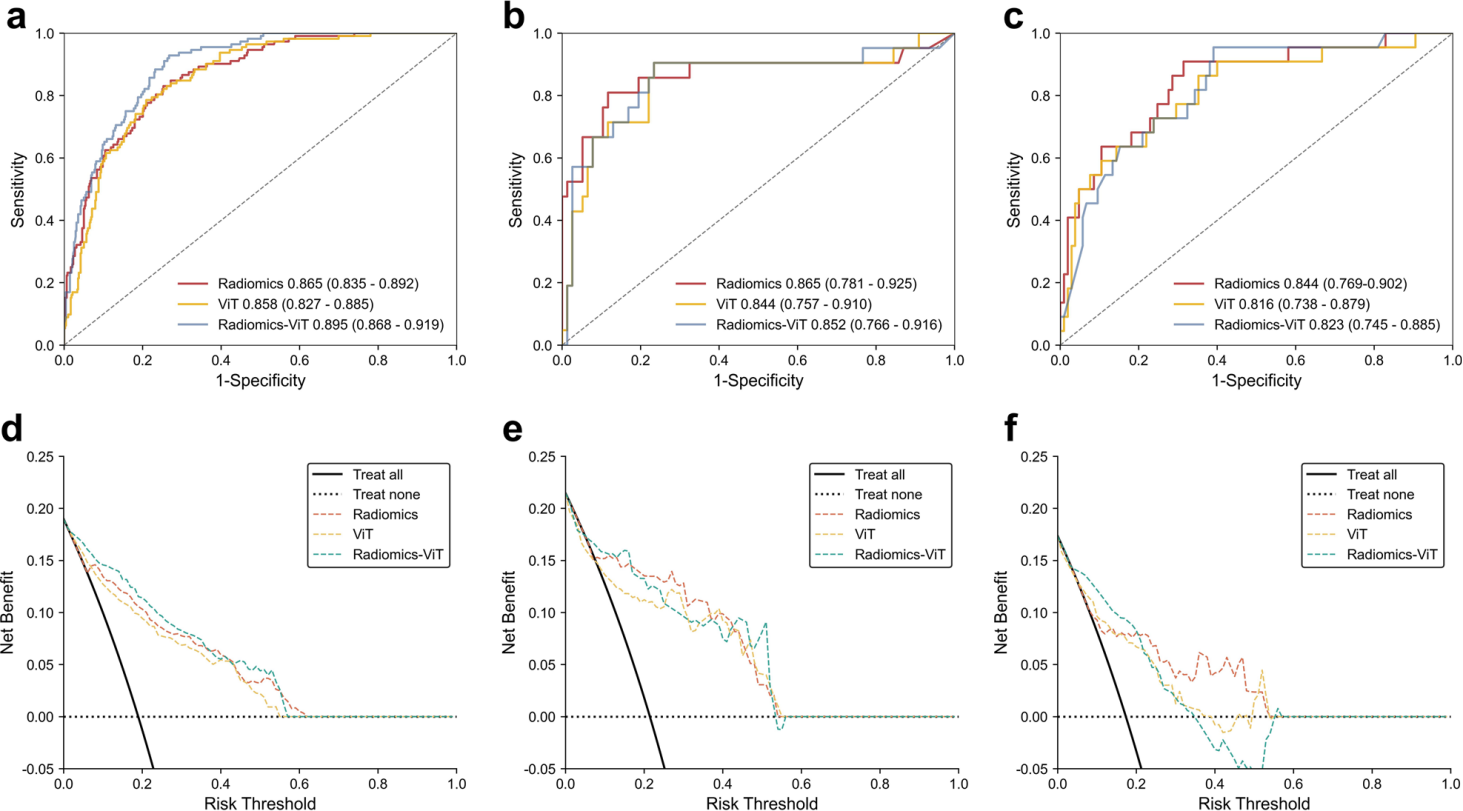
Receiver operating characteristic (ROC) curves for radiomics, ViT, and radiomics-ViT models in training (**a**), internal test (**b**), and external test (**c**) sets; DCA for radiomics, ViT, and radiomics-ViT models in training (**d**), internal test (**e**), and external test (**f**) sets

The receiver operating characteristic (ROC) curves for three models in all datasets are shown in Fig. 4. Across all datasets, the radiomics model yielded numerically higher AUC values than the ViT model, although the differences were not statistically significant. In the training set, the radiomics-ViT model demonstrated a statistically significant improvement over both individual models (*p* < 0.05). However, in the internal and external test sets, the combined model showed performance comparable to the ViT model. Detailed DeLong test results are provided in Supplementary Table S5. The combined model achieved the highest sensitivity (92.8%, 90.5% and 95.4%), while the ViT model exhibited the highest specificity (79.1%, 77.9% and 70.5%). The Brier scores ranged from 0.104 to 0.128 across all models and datasets, indicating good overall calibration without evidence of severe miscalibration. The calibration curves are presented in Supplementary Figure S3. DCA (Fig. 4) demonstrated that the combined model showed higher net benefit than ViT and radiomics models in the training set, while outperforming the single models only within limited threshold probability ranges in the test sets. In subgroup analyses, model performance was compared between the internal and external test sets. Although performance declined from the internal to the external test set in lesions ≤ 20 mm, a statistically significant decrease was observed only for the radiomics model (Supplementary Table S6).

### Model interpretation

**Fig. 5:**
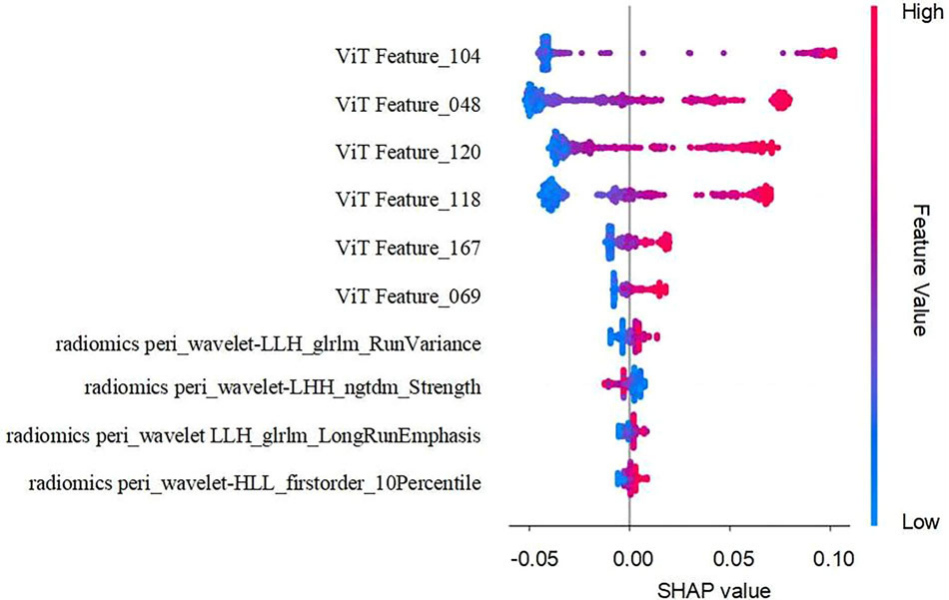
SHAP explanation plot of the radiomics-ViT model

The SHAP explanation for the combined model is shown in Fig. 5. This figure ranks features based on their contribution to the model’s overall predictions. Among the top ten contributing features, the first six were derived from ViT, indicating its substantial contribution to model decision-making. To further enhance the interpretability of the ViT model, attention maps were generated to highlight the image regions most relevant to the prediction (Supplementary Fig. S4).

## Discussion

This retrospective study constructed radiomics, ViT, and radiomics-ViT models based on solid, ground-glass, and peritumoral components for predicting VPI in LUAD ≤ 30 mm. All models achieved AUCs greater than 0.8, with no significant performance decline in the external test set. The radiomics-ViT model showed improved performance in the training set. These findings suggest that radiomics and ViT provide complementary information in identifying VPI. Integration of multiple intratumoral components and peritumoral regions contributes to robust prediction, supporting its potential clinical value.

Previous studies have demonstrated the importance of CT attenuation in assessing high-risk factors. Solid nodules are more likely to invade the pleura than subsolid nodules, while the presence of ground-glass components reduces the likelihood of VPI [25, 26]. Some studies have provided models with information from solid components as additional inputs, improving the prediction of high-risk pathological features such as high-grade histological patterns and spread through air spaces [27, 28]. Moreover, peritumoral features, reflecting tumor-related changes in the surrounding tissues, play an important role in assessing tumor aggressiveness [16, 20]. Therefore, we provided models with solid, ground-glass, and peritumoral regions separately to preserve distinctive characteristics of each component while enabling complementary information integration.

SHAP analysis provided model interpretability. ViT features, extracted from and aggregated across solid, ground-glass, and peritumoral components, contributed most to VPI prediction. Separately extracting features from these regions allows the model to preserve component-specific information and prevents the dilution of biologically meaningful signals. Attention maps assigned higher weights to regions at some interfaces between different tumor components. Some peritumoral radiomics features also showed high contributions, reflecting a spatial relationship between the tumor and the pleura. Among them, Wavelet-LLH_glrlm_RunVariance ranked first. This feature quantifies the variability of consecutive gray levels along a specified direction. A higher RunVariance value reflects greater heterogeneity in the peritumoral gray-level distribution, suggesting more aggressive tumor behavior and microenvironmental changes. These characteristics support the utility of this radiomics feature in predicting high-risk pathological outcomes associated with VPI.

In subgroup analyses, models demonstrated good performance in the ≤ 20 mm group in both the training and internal test sets (AUCs > 0.85). Performance declined in the external test set, although this decrease reached statistical significance only for the radiomics model. This decline may be attributed to inter-center heterogeneity, including differences in imaging protocols and patient populations. Small lesions inherently contain fewer voxels, which may limit the amount of discriminative information available for model training. In contrast, the 20–30 mm subgroup showed stable AUCs across all datasets, indicating greater robustness and clinical applicability in larger tumors. These findings indicate that future studies should consider targeted modeling or increased sample sizes for small lesions to enhance clinical utility.

Notably, clinical decision-making in real-world settings is multifactorial, ranging from patient-specific characteristics, such as anesthetic risk and comorbidities, to broader considerations including the availability of postoperative adjuvant therapy and institutional protocols. The Radiomics-ViT model demonstrated the highest sensitivity in detecting VPI, suggesting its potential to support more aggressive treatment strategies, particularly for younger patients with good pulmonary reserve. Although this approach may entail a certain risk of false positives, the overall assessment prioritizes minimizing the risk of undertreatment and potential recurrence. In contrast, the ViT model exhibited the highest specificity, which may help avoid overtreatment, particularly in resource-constrained settings or patients with limited physiological reserves.

There are some limitations to this study. First, clinical characteristics, hematologic biomarkers, and CT semantic features were not incorporated. Future work will add these factors to further enhance model performance and applicability. Second, the threshold for segmentation was based on previous literature and observational experience [28–30], rather than being derived via systematic internal optimization. Finally, pathological characteristics of the regions emphasized by attention maps, particularly at tumor component interfaces, were not evaluated in a direct imaging-pathology precision registration. Future studies could systematically compare these regions on a slice-by-slice basis.

In conclusion, a multi-component feature extraction and modeling approach can effectively predict VPI. The multi-component radiomics–ViT model demonstrated high sensitivity for VPI prediction in LUAD ≤ 30 mm, highlighting its potential as a valuable tool for preoperative treatment planning and prognostic evaluation.

## Supplementary information

ELECTRONIC SUPPLEMENTARY MATERIAL

## Data Availability

The datasets generated or analyzed during the study are available from the corresponding author upon reasonable request.

## Abbreviations

AUC: Area under the curve
DCA: Decision curve analysis
DL: Deep learning
LUAD: Lung adenocarcinoma
ROI: Region of interest
SHAP: Shapley additive explanations
ViT: Vision transformer
VPI: Visceral pleural invasion
