# Supplementary material for: Multi-component radiological model based on intratumoral CT threshold segmentation for predicting visceral pleural invasion in lung adenocarcinoma ≤ 30 mm
Source: Insights Imaging. 2026 Jul 24;17:194. doi: 10.1186/s13244-026-02351-z (PMC13400552; doi:10.1186/s13244-026-02351-z)
Supplement: Supplementary file 1 — ELECTRONIC SUPPLEMENTARY MATERIAL [file 13244_2026_2351_MOESM1_ESM.pdf]

Multi-component Radiological Model Based on Intratumoral CT Threshold Segmentation for Predicting Visceral Pleural Invasion in Lung Adenocarcinoma ≤30 mm

ELECTRONIC SUPPLEMENTARY MATERIAL

Table S1. CT scanner parameters in center 1

Manufacturer's Model Name	Siemens Healthineers					United Imaging				GE Healthcare	Canon Medical Systems Corporation	
	SOMATOM Emotion 16 16-slice spiral CT scanner	SOMATOM Definition AS 128-slice spiral CT scanner	SOMATOM Definition 64-slice spiral CT scanner	SOMATOM go.ALL 64-slice spiral CT scanner	SOMATOM Definition AS+ 128-slice spiral CT scanner	uCT760 128-slice spiral CT scanner	uCT780 160-slice spiral CT scanner	uMI780 160-slice spiral CT scanner	uCT550 80-slice spiral CT scanner	Discovery VCT 64-slice spiral CT scanner	TOSHIBA AquillonCX 128-slice spiral CT scanner	TOSHIBA AquillonOne 320-slice spiral CT scanner
**Number of patients**	219	17	4	75	15	61	65	85	17	112	11	8
**Tube voltage (kVp)**	130	120	120	120	120	120	120	120	120	120	120	120
**Tube current (mA)**	Automatic											
**Slice Thickness (mm)**	1	1	1	1	1	1	1	0.625	1	0.625	1	1
**Single collimation width (mm)**	0.6	0.6	0.6	0.7	0.6	0.5	0.5	0.5	0.55	0.625	0.5	0.5
**Matrix**	512×512											
**Spiral Pitch Factor**	1.2	1	0.9	1	0.95	0.935/1.088	0.988	0.925	1.075	1.375	1.484	0.828
**Rotation direction**	CW											
**Convolution kernel**	B70s	B30f	B70f	Br64f	B60f	B_VSHARP_B/C	B_VSHARP_C	B_VSHARP_C	B_SOFT_B	LUNG	FC52	FC56
**Window center (HU)**	-600	-600	-600	-550	-600	-600	-600	-600	-600	-700	-600	-600

**Window width (HU)**	1000	1000	1000	1000	1000	1000	1000	1000	1000	1500	1000	1000
**Exposure Time (s) per rotation**	0.6	0.5	0.5	0.5	0.5	0.519	0.506	0.54	0.651	0.4	0.5	0.6
**Photometric Interpretation**	MONOCHROME 2											

Table S2. CT scanner parameters in center 2

Manufacturer's Model Name	Philips		United Imaging	GE Healthcare	
	Brilliance iCT 256-slice spiral CT scanner	Ingenuity 128-slice spiral CT scanner	uCT 510 16-slice spiral CT scanner	Revolution 256-slice spiral CT scanner	Discovery CT750 HD 64-slice spiral CT scanner
**Number of patients**	31	32	21	35	8
**Tube voltage (kVp)**	120	120	120	120	120
**Tube current (mA)**	496	328	Automatic	Automatic	Automatic
**Slice Thickness (mm)**	1				
**Single collimation width (mm)**	0.625	0.625	0.6	0.625	0.625
**Matrix**	1024×1024	1024×1024	1024×1024	512×512	512×512
**Spiral Pitch Factor**	0.992	0.984	0.937	0.984	0.984
**Rotation direction**	CW				
**Convolution kernel**	C	C	B_SOFT_B	STANDARD	STANDARD
**Window center (HU)**	-520	-520	-520	-520	-500
**Window width (HU)**	1450	1450	1450	1450	1500
**Exposure Time(s) per rotation**	0.5	0.76	0.64	0.7	0.6
**Photometric Interpretation**	MONOCHROME 2				

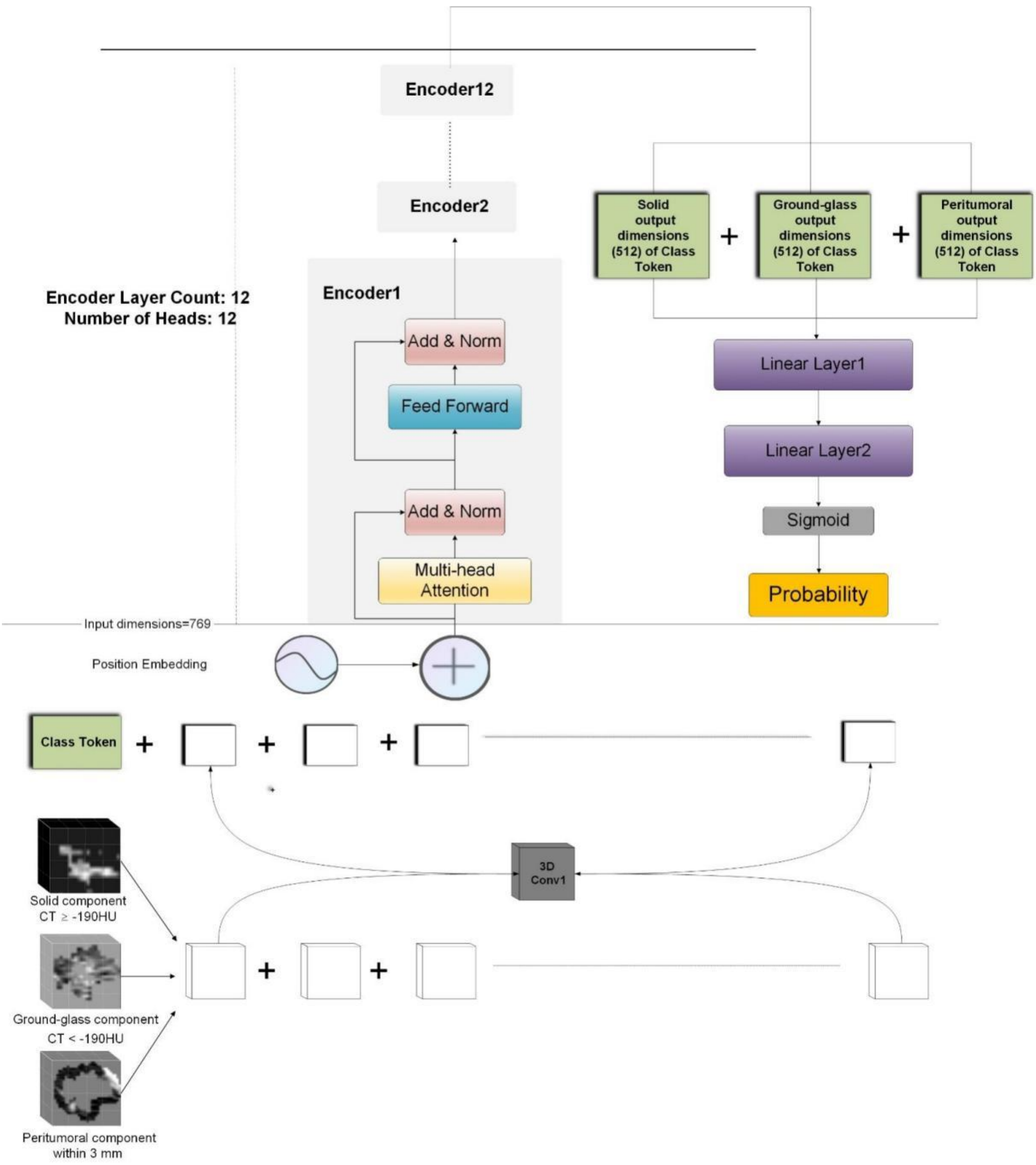

Figure S1: The detailed structure of ViT model

Table S3. LASSO Feature Selection for Radiomics Model

Region	Image Type (Original/Wavelet-Transformed) _ Feature Category_Feature Name	Coefficients
Ground-glass (CT < -190 HU)	original_gldm_DependenceNonUniformityNormalized	7.97E-03
	wavelet-HHH_gldm_DependenceEntropy	-8.40E-03
	wavelet-LLL_glcm_DifferenceAverage	4.55E-04
Solid (CT ≥ -190 HU)	original_glcm_Imc2	-2.90E-03
	wavelet-LLH_glrlm_LongRunHighGrayLevelEmphasis	9.27E-03
	wavelet-LLH_glrlm_RunVariance	4.05E-02
	wavelet-HLL_firstorder_Kurtosis	2.05E-02
	wavelet-LLL_firstorder_Kurtosis	3.24E-03
	wavelet-LLL_firstorder_Skewness	-2.47E-02
	wavelet-LLL_glszm_GrayLevelNonUniformity	1.22E-02
Peritumoral (within 3 mm)	original_gldm_DependenceEntropy	1.25E-02
	wavelet-LLH_firstorder_Skewness	8.92E-03
	wavelet-HLL_firstorder_Mean	3.75E-03
	wavelet-LLL_firstorder_RobustMeanAbsoluteDeviation	1.56E-02
	wavelet-LLL_gldm_LargeDependenceHighGrayLevelEmphasis	4.83E-03

LASSO: Least Absolute Shrinkage and Selection Operator

Table S4. LASSO Feature Selection for Radiomics-ViT Model

Region		Image Type (Original/Wavelet-Transformed) _ Feature Category_Feature Name	Coefficients
Radiomics	Ground-glass (CT < -190 HU)	wavelet-LHH_glcm_ClusterShade	4.97E-03
		wavelet-HLH_glcm_Correlatio	1.23E-03
	Solid (CT ≥ -190 HU)	Wavelet-LLH_gldm_LargeDependenceHighGrayLevelEmphasis	1.07E-02
	Peritumoral (within 3 mm)	wavelet-LLH_firstorder_Skewness	3.18E-03
		wavelet-LLH_glrlm_LongRunEmphasis	5.38E-04
		wavelet-LLH_glrlm_RunVariance	4.59E-03
		wavelet-LLH_ngtdm_Busyness	7.36E-03
		wavelet-LHH_firstorder_Maximum	-1.86E-03
		wavelet-HLL_firstorder_10Percentile	9.63E-03
		wavelet-LHL_gldm_LargeDependenceHighGrayLevelEmphasis	1.25E-02
		wavelet-LHH_gldm_SmallDependenceHighGrayLevelEmphasis	-8.31E-04
		wavelet-LHH_ngtdm_Strength	-1.50E-03
ViT		Feature_048	3.01E-02
		Feature_069	1.80E-02
		Feature_104	3.67E-02
		Feature_118	7.28E-02
		Feature_120	7.75E-03
		Feature_161	1.76E-02
		Feature_167	5.70E-03

LASSO: Least Absolute Shrinkage and Selection Operator

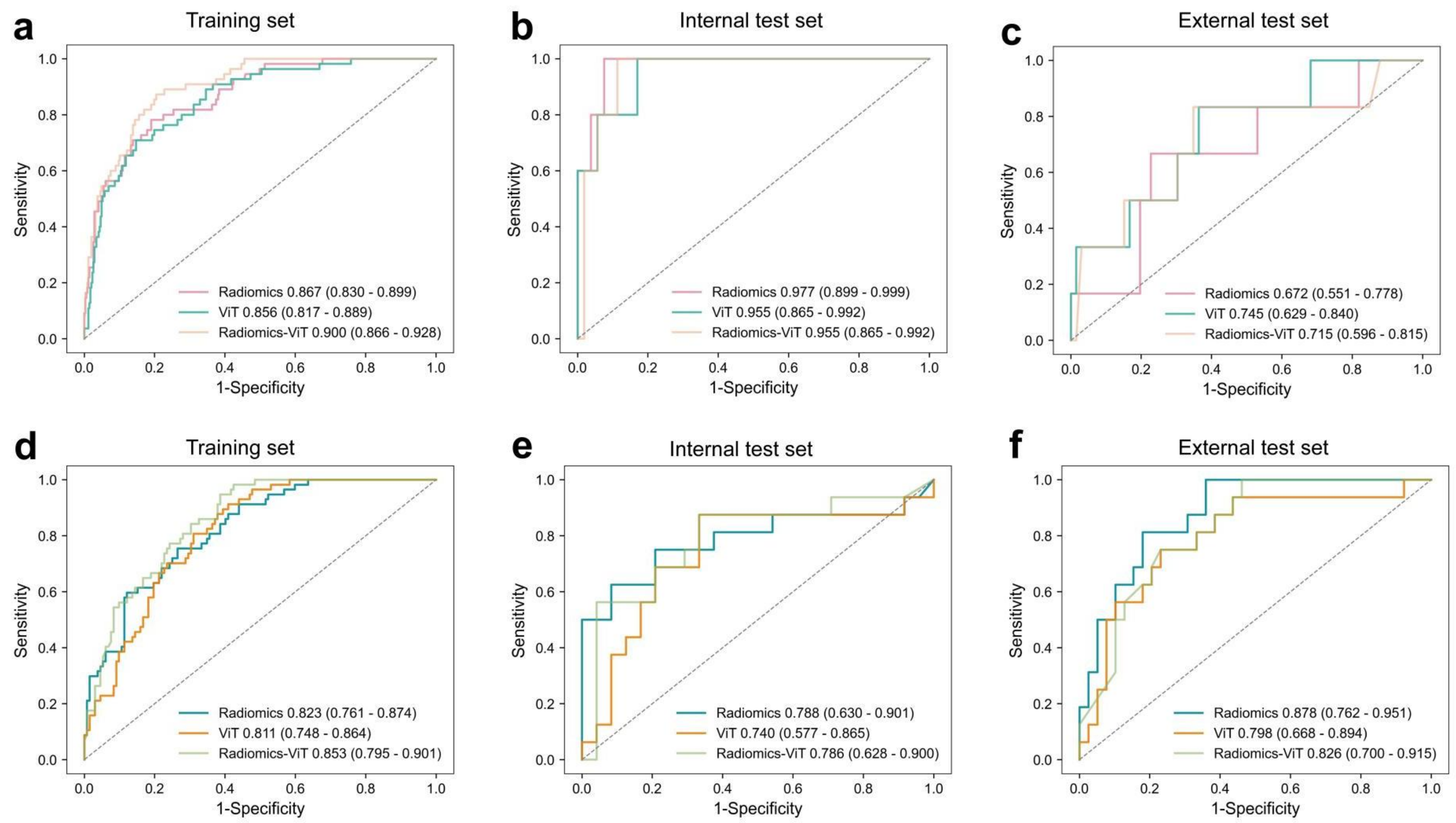

Figure S2. ROC curves for the ≤20 mm subgroup in the training (a), internal test (b), and external test (c) sets, and for the 20-30 mm subgroup in the training (d), internal test (e), and external test (f) sets.

Table S5. Comparison of model AUCs using the DeLong test

	Difference between areas	Z statistics	SE	95% CI	P value
Training set					
Radiomics vs. ViT	0.00746	0.679	0.0110	-0.0141 to 0.0290	0.4973
Radiomics vs. Radiomics-ViT	0.0301	2.736	0.0110	0.00853 to 0.0516	0.0062
ViT vs. Radiomics-ViT	0.0375	6.709	0.00559	0.0266 to 0.0485	<0.0001
Internal test set					
Radiomics vs. ViT	0.0204	0.955	0.0214	-0.0215 to 0.0623	0.3398
Radiomics vs. Radiomics-ViT	0.0127	0.552	0.0230	-0.0324 to 0.0577	0.5812
ViT vs. Radiomics-ViT	0.00773	0.723	0.0107	-0.0132 to 0.0287	0.4698
External test set					
Radiomics vs. ViT	0.0281	0.737	0.0382	-0.0467 to 0.103	0.4613
Radiomics vs. Radiomics-ViT	0.0214	0.828	0.0259	-0.0293 to 0.0722	0.4079
ViT vs. Radiomics-ViT	0.00671	0.245	0.0274	-0.0470 to 0.0604	0.8064

AUC, area under the curve; CI, confidence interval.

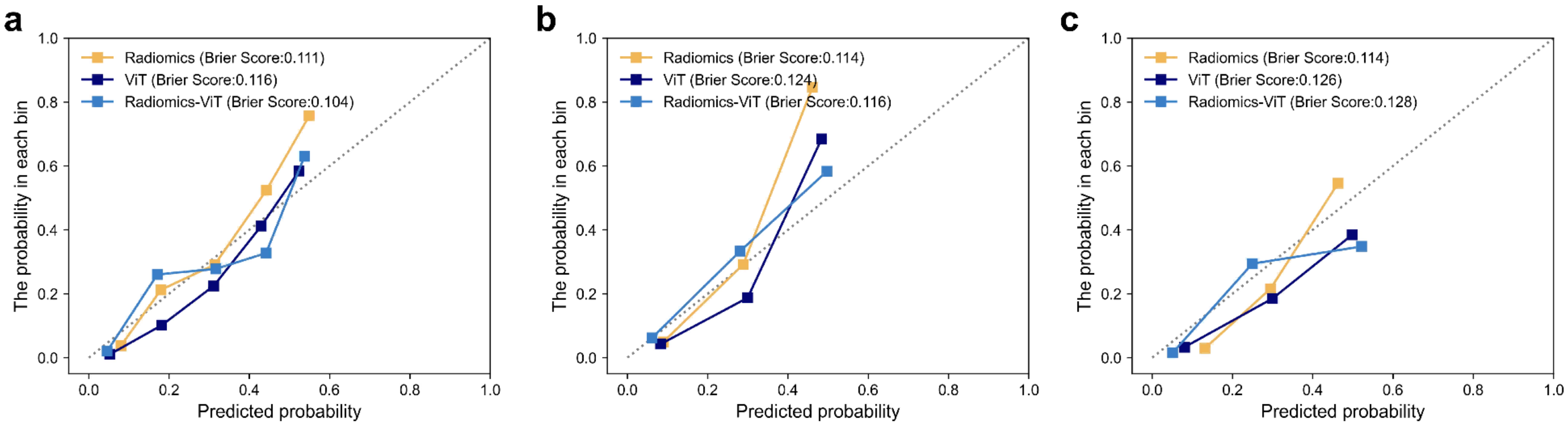

Figure S3. Calibration curves. Radiomics, ViT, and radiomics-ViT models in training set (a), internal test set (b), and external test set (c).

Table S6. Δ AUC between internal and external test sets stratified by lesion size

Subgroup	Data	Model	ΔAUC (95% CI)	P value
≤20 mm	Internal test set (N=58; Pos/Neg=5/53) vs. External test set (N=72; Pos/Neg=6/66)	Radiomics	0.306 (0.062 to 0.588)	0.012
		ViT	0.210 (-0.014 to 0.470)	0.070
		Radiomics-ViT	0.240 (-0.008 to 0.552)	0.066
20-30 mm	Internal test set (N=40; Pos/Neg=16/24) vs. External test set (N=55; Pos/Neg=16/39)	Radiomics	-0.090 (0.303 to 0.082)	0.356
		ViT	-0.058 (-0.284 to 0.147)	0.606
		Radiomics-ViT	-0.040 (-0.242 to 0.146)	0.680

AUC, area under the curve; CI, confidence interval

N denotes the number of lesions. Pos/Neg indicates the number of VP-present and VPI-absent lesions in each dataset.

ΔAUC represents the difference in AUC between the internal and external test sets.

95% CI was estimated using 1000 bootstrap resamples.

P value tests whether the observed change in AUC differs significantly from zero.

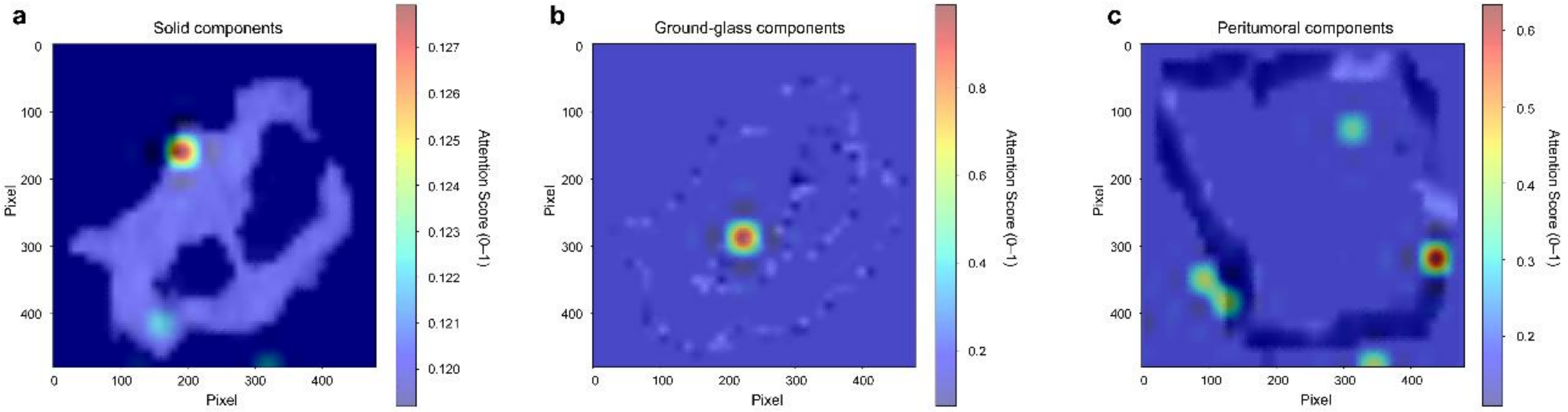

Figure S4. Attention maps of a positive sample from the ViT model. a, b, and c represent the solid, ground-glass, and peritumoral components of the lesion, respectively. The vertical color bars on the right denote attention intensity, ranging from blue (low) to red (high). Regions with higher attention values indicate areas to which the model assigned greater importance during prediction.
